# CD4^+^ICOS^+^Foxp3^+^: a sub-population of regulatory T cells contribute to malaria pathogenesis

**DOI:** 10.1186/s12936-022-04055-3

**Published:** 2022-02-02

**Authors:** Rubika Chauhan, Vikky Awasthi, Reva Sharan Thakur, Veena Pande, Debprasad Chattopadhyay, Jyoti Das

**Affiliations:** 1 Parasite-Host Biology, National Institute of Malaria Research, Sector-8, Dwarka, New Delhi, 110077 India; 2 Biotechnology Department, Kumaun University, Nainital, India; 3 ICMR Virus Unit, ID and BG Hospital, Kolkata, 700010 India; 4 ICMR-National Institute of Traditional Medicine (NITM), Belagavi, 590010 India

**Keywords:** Malaria, *Plasmodium berghei*, *Plasmodium chabaudi*, *Plasmodium yoelii*, IL-10, Regulatory T cell, Lethal, Non-lethal

## Abstract

**Background:**

Regulatory T cells are known to play a key role to counter balance the protective immune response and immune mediated pathology. However, the role of naturally occurring regulatory cells CD4^+^CD25^+^Foxp3^**+**^ in malaria infection during the disease pathogenesis is controversial. Beside this, ICOS molecule has been shown to be involved in the development and function of regulatory T cell enhance IL-10 production. Therefore, possible involvement of the ICOS dependent regulatory CD4^+^ICOS^+^Foxp3^**+**^ T cells in resistance/susceptibility during malaria parasite is explored in this study.

**Methods:**

5 × 10^5^ red blood cells infected with non-lethal and lethal parasites were inoculated in female Balb/c mice by intra-peritoneal injection. Infected or uninfected mice were sacrificed at early (3rd day post infection) and later stage (10th day post infection) of infection. Harvested cells were analysed by using flow cytometer and serum cytokine by Bioplex assay.

**Results:**

Thin blood films show that percentages of parasitaemia increases with disease progression in infections with the lethal malaria parasite and mice eventually die by day 14th post-infection. Whereas in case of non-lethal malaria parasite, parasitaemia goes down by 7th day post infection and gets cleared within 13th day. The number of CD4^+^ ICOS^+^ T cells increases in lethal infection with disease progression. Surprisingly, in non-lethal parasite, ICOS expression decreases after day 7th post infection as parasitaemia goes down. The frequency of CD4^+^ICOS^+^FoxP3^+^ Tregs was significantly higher in lethal parasitic infection as compared to the non-lethal parasite. The level of IL-12 cytokine was remarkably higher in non-lethal infection compared to the lethal infection. In contrast, the level of IL-10 cytokines was higher in lethal parasite infection compared to the non-lethal parasite.

**Conclusion:**

Taken together, these data suggest that lethal parasite induce immunosuppressive environment, protecting from host immune responses and help the parasite to survive whereas non-lethal parasite leads to low frequencies of Treg cells seldom impede immune response that allow the parasite to get self-resolved.

**Graphical Abstract:**

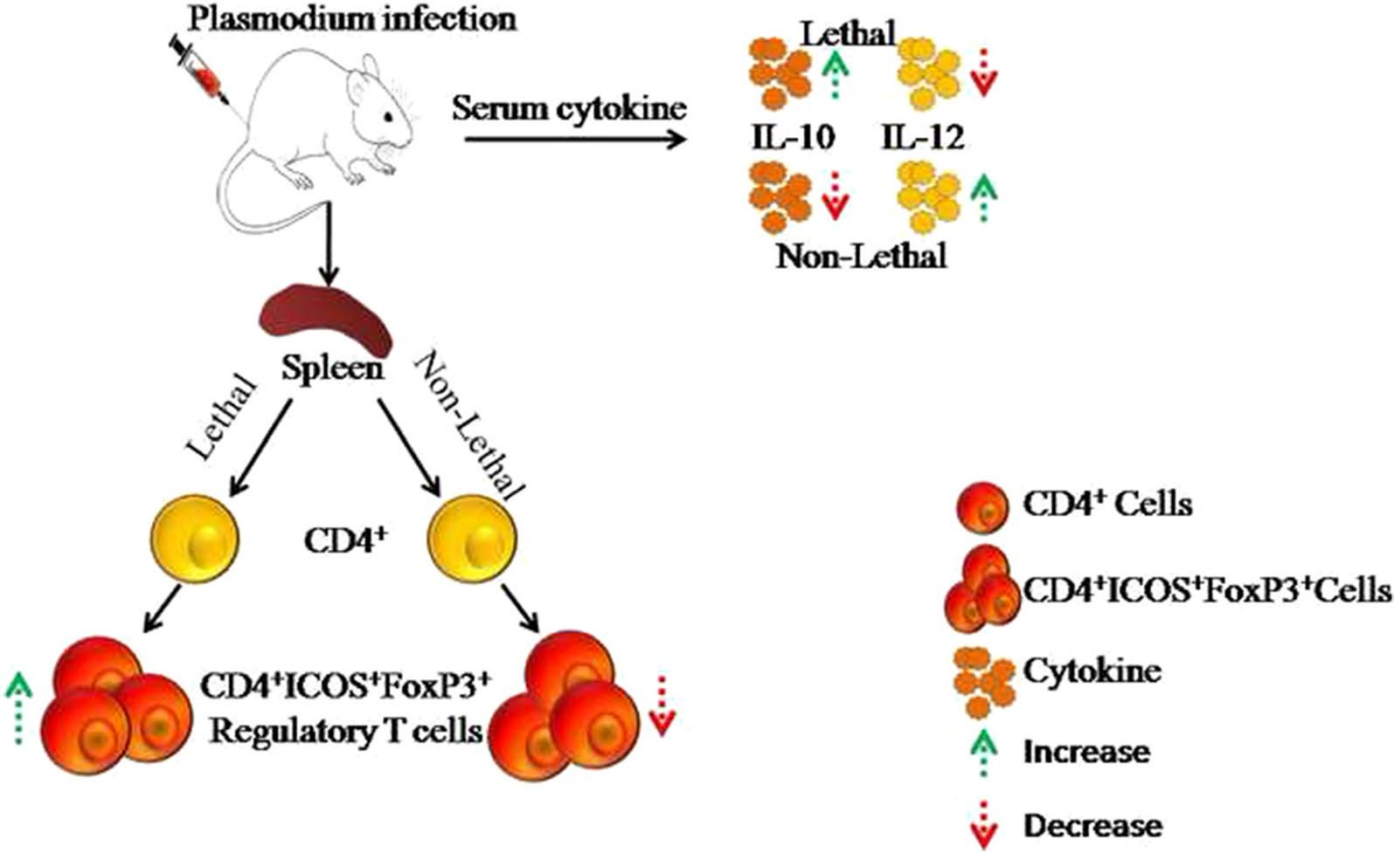

**Supplementary Information:**

The online version contains supplementary material available at 10.1186/s12936-022-04055-3.

## Background

Malaria is amongst one of the major infectious disease worldwide like, AIDS and tuberculosis [1, 2]. The 2020 World Malaria Report estimated that there were 229 million cases of malaria in 2019, compared with 238 million cases in 2018 [3]. Even though success of various public health measures made, malaria is still a challenging disease in developing countries like South East Asia, South America and Africa [4]. Malaria disease is characterized by inflammation and massive infiltration of immune cells in spleen resulting into splenomegaly [5]. Besides this, accumulation of malarial pigment, haemozoin (Hz) during infection results in ineffective erythropoiesis leading to severe malarial anaemia and dysregulation of the inflammatory mediators [6]. However, the host immune response acts differently with different species of *Plasmodium* parasites [7]. It is well reported that blood-stage infection with rodent parasites, such as *Plasmodium yoelii*, *Plasmodium berghei* and *Plasmodium chabaudi* induce potent inflammatory response and leads to various manifestations of experimental malaria [8].

Inducible co-stimulatory molecule (ICOS), member of immunoglobin (Ig) family of co-receptor molecules has been identified as a new molecule responsible for adaptive immune response. ICOS is expressed on activated CD4 and CD8 T lymphocytes. Several studies have evaluated the role of ICOS in T cell proliferation and immune response [9–11]. In contrast, CD28 being homologous to ICOS is expressed constitutively [12, 13]. Several reports using ICOS deficient mice have revealed that T cells exhibit a proliferation defect in vitro and in vivo as well when compared with T cells of wild type CD4 T cells making them less efficient in mounting T cell response [10] and induce AKT pathway to regulates effector immune responses [14].

Regulatory T cell (Treg) subset of T helper cells is characterized by expression of Forkhead box p3 (Foxp3) transcription factor. It mitigates the immune system through secretion of IL-10. Several studies have indicated the critical role of T regs cell in maintaining homeostasis of immune system and development of immune tolerance. Regulatory T cell mediated suppression prevents the immuno-pathology associated with excessive inflammation during infection. Thus, regulatory T cells regulate the adaptive immune response and help in maintenance of homeostasis. Some reports claim that regulatory T cells induced during malaria infection assist in parasite clearance and prevent from immune-pathology while other reports provide evidences that regulatory T cells facilitate parasite persistence by down-regulating effector immune response [15, 16]. Thus the role of these induced regulatory T cells remains debatable.

The vital role of Foxp3 in mounting effective immune response is associated with the production of regulatory cytokine IL-10. Cytokines are known to play an important role in determining the outcome of malaria infection by regulating both cellular and humoral immune responses. Interleukin 10 (IL-10) is also well-known anti-inflammatory and immunosuppressive in nature. A number of pathogens have been shown to induce the production of IL-10 for their survival and disease persistent [17]. Following infection, the increased expression of IL-10 further inhibits the activity of Th1 cells, NK cells, and macrophages which are crucial for pathogen clearance. The regulatory cytokine IL-10, secreted by Tregs has been associated with modulation of the disease severity and hinders the development of protective immunity [18, 19]. IL-10 has been found detrimental in various pathogens, including *Leishmania* spp. [20], *Candida* spp*.* [21], *Mycobacterium tuberculosis* [22, 23], *Bordetella* spp*.* [24] and HIV[25]. Studies have shown that IL-10 suppresses the immune system in *P. berghei* induced malarial infection and helps in diseases exacerbation thus facilitating pathogenesis of malaria infection [5, 26, 27]. After a successful immune response, a balance is obligatory between protection and immuno-pathology, and IL-10 is considered as crucial turning point to establish this balance [28, 29]. In rodent malaria, *P. berghei* and *P. yoelii* strain 17XL infection is considered lethal to the host [30, 31], whereas infection with *P. chabaudi* and *P. yoelii* strain 17XNL is considered as non-lethal to the host [31, 32]. The mechanism, how effector host immune system is able to clear the infection with non-lethal parasite while lethal parasite takes over the immune response and results in immune pathology still remains to be elucidated. To understand the differential effector immune responses in these species of *Plasmodium* infection, the expression of activation markers (ICOS), and regulatory T cells expressing the both markers (CD4^+^ICOS^+^Foxp3^+^) were compared. Furthermore, levels of IFN-γ, IL-12 and IL-10 were also analysed during the infection in Balb/c mice to further re-confirm the results.

## Methods

### Animals

Female Balb/c (n  = 9 for each malaria parasite) specific pathogen free mice at 7–9 weeks of age were purchased from the Central Drug Research Institute (CDRI; Lucknow, India) and maintained at NIMR for experiment. Animals were approved by institute ethical committee and Reference Approval Number is IAEC/NIMR/2012-3.

### Parasite infection in mice

Cryopreserved lethal (*P. berghei* and *P. yoelii* 17XL*)* and non-lethal (*P. chabaudi* and *P. yoelii* 17XNL) (obtained from the parasite bank of the National Institute of Malaria Research, New Delhi, India). *Plasmodium berghei*, *P. yoelii* 17XL, *P. chabaudi* and *P. yoelii* 17XNL were passaged once through mice before use in experimental animals. Mice were infected with 5 × 10^5^ syngeneic parasitized erythrocytes (pRBCs) by intra-peritoneal injection as described elsewhere [5].

### Determination of parasitaemia

To determine the extent of infection, blood smears were prepared from tail vein at various time points as indicated in figure legends and stained with Giemsa. Parasitaemia was determined by counting the percentage of infected cells per 5000 RBCs per slide, as described in WHO SOP (reference number: WHO/HTM/GMP/MM/SOP/2016.09).

### Splenocyte preparation

Single cell suspensions of splenocytes were prepared by smashing spleens of infected and control mice. RBCs were lysed with RBC lysis buffer (0.15 M NH_4_Cl, 10 mM KHCO_3_, 0.1 mM Na_2_EDTA) and the cells were washed and then re-suspended in RPMI 1640 medium (Gibco-life technologies) supplemented with 10% FBS (Hyclone, USA) and 100 IU/ml penicillin–streptomycin (Gibco-BRL).

### Flow cytometer

For flow cytometric analyses, on various time points as indicated in figure legends, spleen cells from mice were re-suspended in 30 µl of FACS buffer (PBS, 2% FCS) and surface-stained at 4 °C with anti-mouse CD4 (APC, Clone: RM4-5), CD8 (FITC, Clone: 53–6.7), CD274 (PE, Clone: J43). All antibodies were purchased from BD biosciences. Stained cells were acquired with a FACS Fortessa (BD Biosciences) and analysed by FlowJo (Tree star) software.

### Intracellular staining

Splenocytes were activated with 1 µg/ml anti-CD3 and 2 µg/ml of anti-CD28 Brefeldin-A and Monensin (BD Pharmingen) were added at 6 h of incubation at a final concentration of 1 µg/ml to inhibit cytokine secretion. For cell-surface staining, cells were suspended in staining buffer (PBS, 3% FCS) at a concentration of 10^7^ cells/ml, and 30 µl of suspension was incubated with fluorescent antibodies for 30 min on ice. Cells were washed twice with staining buffer and fixed with 1% para-formaldehyde. For intracellular staining, cells were washed twice with PBS and re-suspended in a permeabilization buffer (Cytofix/Cytoperm kit; BD), and stained with fluorescent conjugated antibodies IL-10 (PE, Clone: JES516E3), Foxp3 (APC, Clone: MF23), IFN-γ (APC, Clone: XMG1.2), CD4 (Percep, Clone: GK1.5) and CD274 (BV-421, Clone: J43). Fluorescence intensity was measured by flow cytometry (FACS Fortessa).

### Serum sample analysis

Serum sample was analysed to check the level of cytokine. Levels of cytokine in serum of infected mice were patterned by multiplex bead array immunoassay using Luminex technology (Bio-Plex; Bio-Rad Laboratories).

### Statistical analysis

All statistical analysis was performed in MS excel 2007. Student’s *t *test was used as indicated in figures and considered significant when p  ≤ 0.05.

## Results

### Parasitaemia and survival

**Fig. 1:**
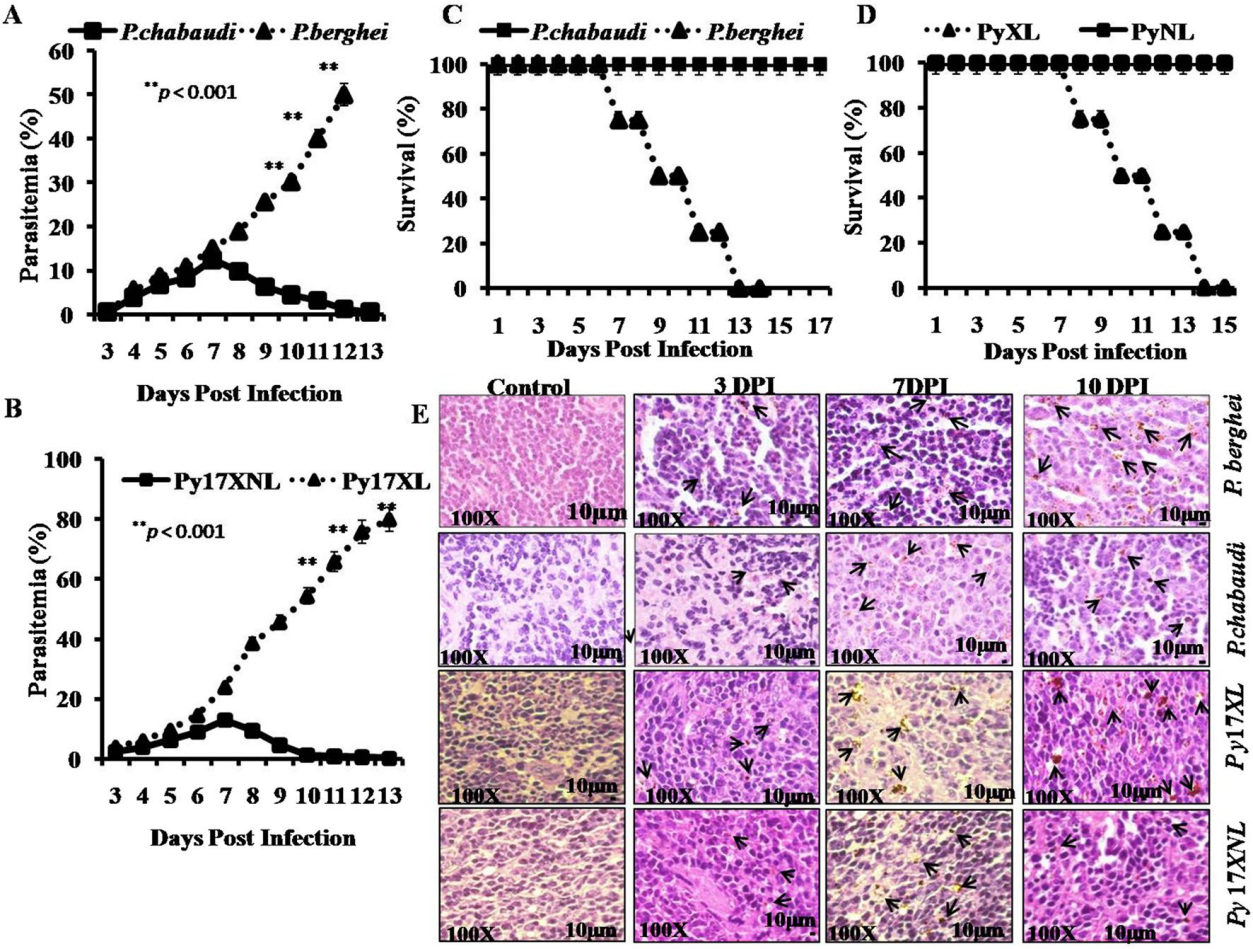
Survival and parasitaemia in lethal and non-lethal infected mice: Balb/c mice were infected with 5 × 10^5^ *P. berghei* and *P. chabaudi* parasitized erythrocytes via intra-peritoneal injection. Malaria parasites infected mice were monitored and parasitaemia was examined. Blood smear were prepared from tail vein at various time points as indicated in the figure and stained with giemsa stains. Parasitaemia was determined by counting the percentage of infected cells per 5000 RBCs per slide. **A** Parasitaemia of *P. berghei* (triangle, n  =  9) and *P. chabaudi* (Square, n  = 9) at different time points. **B** Parasitaemia of *P. yoelii*17XL (Py17XL) (triangle, n  = 9) and *P. yoelii*17XNL (Py17XNL) (Square, n  = 9) at different time points. Statistical significance was determined by Student’s *t *test. **p  < 0.001. **C** Survival rate of *P. berghei* and *P. chabaudi* infected mice. **D** Survival rate of *P. yoelii* 17XL and *P. yoelii* 17XNL infected mice. Nine mice pooled from three independent experiments consisting of three mice in each group. Immunohistochemistry of haemozoin contents in the spleen of Balb/c mice **E** infected with *P. berghei*, *P. chabaudi*, *P. yoelii* 17XL and *P. yoelii* 17XNL at 3, 7 and 10 day post infection. Black arrows indicate the haemozoin content at 100 ×  original magnification

To determine the mode of infectivity of *Plasmodium* species: *P. berghei* and *P. chabaudi* (please note that Pb and Pc are not acceptable abbreviations, while *P. berghei* and *P. chabaudi* are) in vivo, BALB/c mice were used and infected with 5 × 10^5^ syngeneic parasitized erythrocytes (pRBCs) by intra-peritoneal (i.p.). Parasitaemia was determined on every single day till the end of experiment as mentioned in the Fig. 1A. It was observed that animals infected with *P. berghei* exhibited gradual increase in parasitaemia till they die on 13–14th day post infection. Whereas animals infected with *P. chabaudi* displayed a progressive decrease in parasitaemia post 7th day. Parasitic load increased during early time points (3rd day post infection) and attained infectivity percentage of around 12% on 7th day post infection. A drastic decrease in parasitaemia on day 10 post infection was observed. Parasitaemia gets cleared on later stage (13th day post infection). All the animals survived until the termination of the experiment suggesting non-lethal nature of parasite (Fig. 1C).

Further, infectivity of another species of *Plasmodium* parasite, *P. yoelii* 17XL (lethal) and *P. yoelii* 17XNL (non-lethal) in Balb/c mice was determined to ascertain the pattern of infection (Fig. 1B). The result obtained was in consistent as shown in Fig. 1A, C. It was observed that parasitic load in mice infected with *P. yoelii* 17XL increases quickly and all animals died at 9–13 days post infection. On the other hand, *P. yoelii* 17XNL parasitaemia increased gradually until 7 days post infection, followed by decrease in parasitic count and get cleared completely by 13 days post infection. All the infected animals recovered and 100% survival was observed (Fig. 1D).

### Expression of inducible co-stimulatory (ICOS) molecules during the progress of malaria infection

**Fig. 2:**
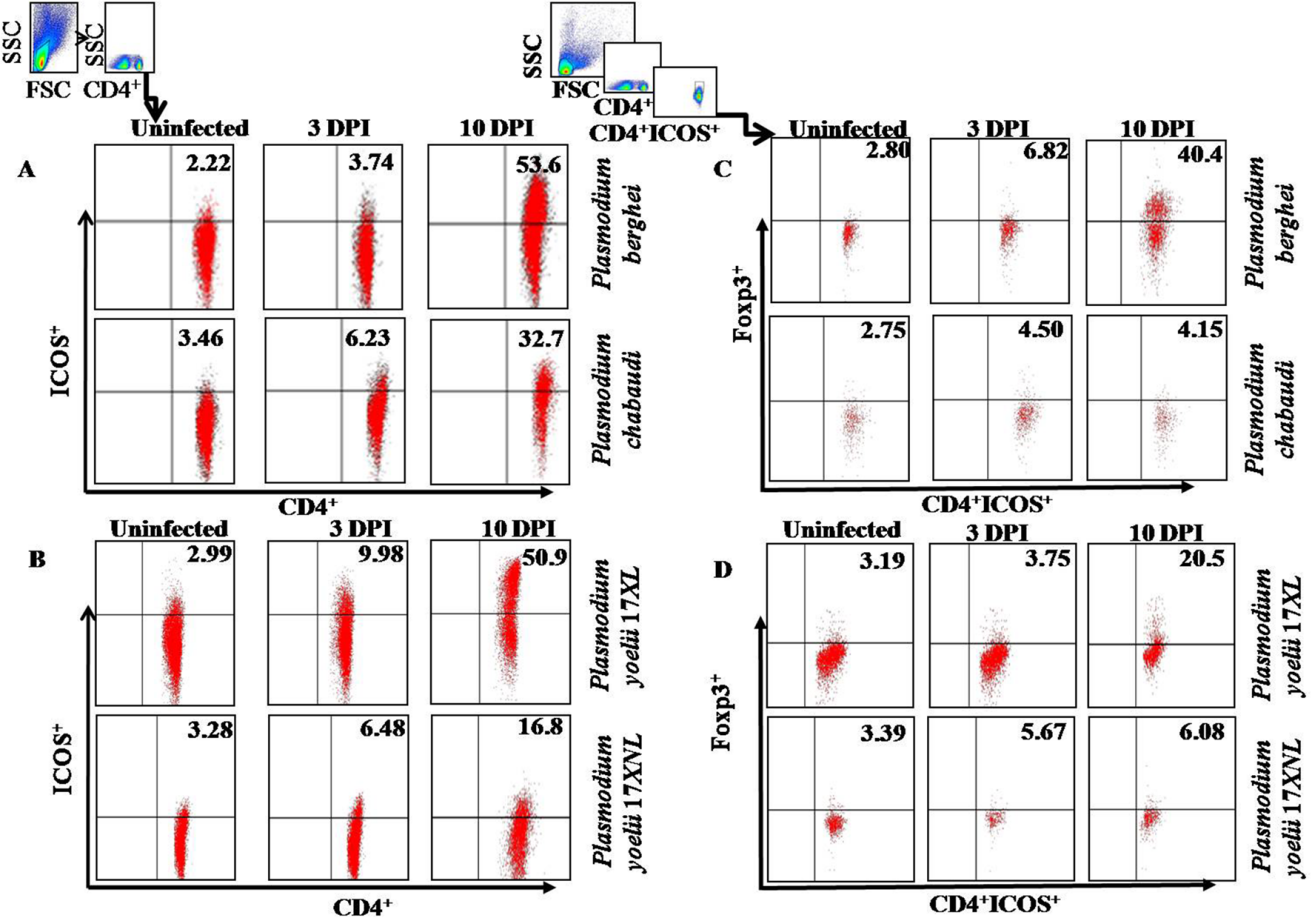
Immune responses in malaria infection with lethal and non-lethal stain of Plasmodium: mice were infected with 5 × 10^5^ *P. berghei*, *P. chabaudi*, Py17XL and Py17XNL -parasitized erythrocytes via intra-peritoneal injection. **A **Splenocytes from infected and control mice were harvested and were stained with antibodies against CD4, and ICOS on 3rd, 10th-day post infection of *P. berghei* and *P. chabaudi*. **B** Splenocytes from infected and control mice were harvested and were stained with antibodies against CD4, and ICOS on 3rd, 10th-day post infection of *P. yoelii* 17XL and *P. yoelii* 17XNL. **C** Determination of Treg cells in malaria infection. Splenocytes were analyzed for Treg by staining with antibodies against CD4^+^ ICOS^+^ and Foxp3^+^cells by flow cytometry. Splenocytes from Pb, Pc infected and control mice were harvested on 3rd and 10th days post infection. **D** Expression of Treg cells in *P. yoelii* 17XL and *P. yoelii* 17XNL malaria infection. Splenocytes from Py17XL, Py17XNL infected and control mice were harvested on 3rd and 10th days post infection. Data is shown from one of the three independent experiments consisting of three mice in each group

To have an insight of immune activation following parasite infection we determined the expression of co-stimulatory molecule responsible for the T cell mediated immune response. ICOS molecule is expressed on CD4^+^ T cells upon activation. To understand the function of this molecule induced in animals infected with lethal and non-lethal malaria, splenocytes of infected animals at early (3rd day) and later stage (10th day) of infection were harvested and the expression of ICOS on CD4 ^+^ T cells using flow cytometer was determined. It was observed that number of CD4^+^ T cells expressing ICOS gradually increased in both sets of animals infected with either lethal or non-lethal malaria parasite. However, frequency of CD4^+^ ICOS^+^ T cells decreased after 7th day post infection (Additional file 1: Figs. S1, S2), in animals infected with non-lethal malaria parasite as compared to lethal parasite with gradual increase in the number of these cells as shown in Fig. 2A, B. These observations indicate that lethal and non-lethal malaria parasite differentially regulates the expression of co-stimulatory molecules (ICOS) on CD4^+^ T cells.

### Differential expression of CD4^+^ICOS^+^Foxp3^+^T regulatory cells in lethal and non-lethal

It is well known that ICOS mediates immune regulation by facilitating the expression of nuclear transcription factor, Forkhead box P3 (FoxP3) which is the defining property of Treg development and function. CD4^+^CD25^+^Foxp3^+^are known as naturally occurring T regulatory cells [33]. In malaria, regulatory T cells (Treg) are the important contributors of immune modulation during infections. The role of these cells has been investigated extensively, though the conclusion is yet controversial. Therefore, to pinpoint their role in malaria, animals were infected with lethal and non-lethal parasite of malaria. Upon intracellular staining of Foxp3, it was found that infection with lethal malaria parasite induced significantly higher frequencies of CD4^+^ICOS^+^Foxp3^+^Treg cells with nearly 50% of CD4^+^ICOS^+^ T cells expressed Foxp3 by day 10th post infection. However, frequencies of CD4^+^ICOS^+^Foxp3^+^ Treg cells during infection with non-lethal parasite remains significantly low (approximately 4%) by day 10th post infection (Fig. 2C, D). These results suggest  ~  ten-fold difference in the frequencies of regulatory T cells at that time point which might decide the fate of host survival. Thus, immune response tends to become more towards immuno-suppressive with lethal malaria parasite with higher numbers of CD4^+^ICOS^+^Foxp3^+^regulatory T cells whereas with the non-lethal parasite no significant CD4^+^ICOS^+^Foxp3^+^regulatory T cell population was observed.

### Cytokine profile during infection with lethal and non-lethal *Plasmodium* parasite

**Fig. 3:**
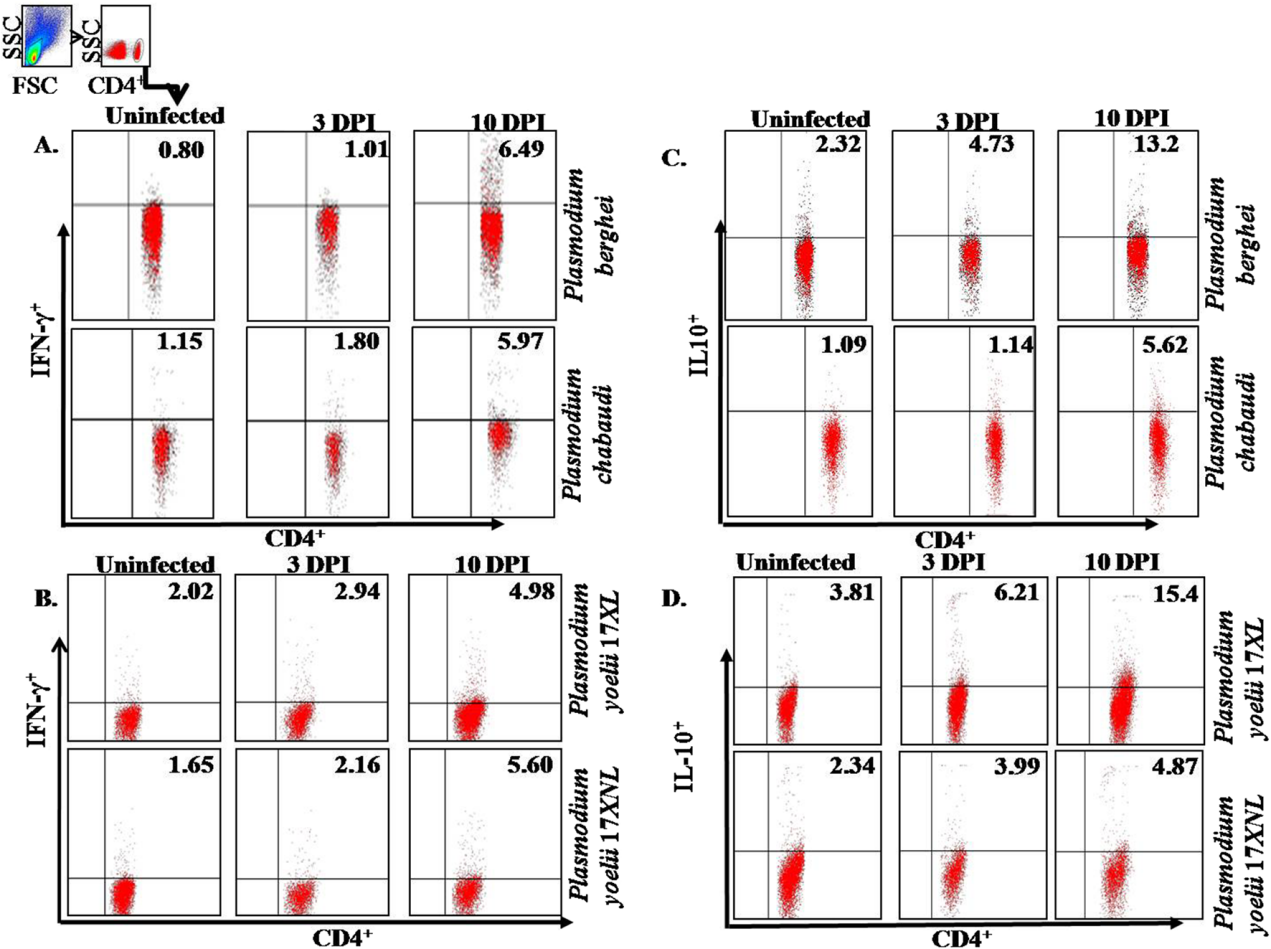
Production of cytokines in malaria infection: IL-10 and IFN-γ producing CD4^+^ cells were checked by flow cytometer during the course of malaria infection on 3rd, and 10th days post infection. IFN-γ expressing CD4^+^ cells during the course of lethal and non-lethal malaria infection (**A**, **B**) and IL-10 expressing CD4^+^ Tcells during the course of lethal and non-lethal malaria infection (**C**, **D**). Data is shown from one of the three independent experiments consisting of three mice in each group

Further investigation was performed to demonstrate the effect of difference in lethality of parasite on immuno-modulation. For this, cytokines secreted by T cells were determined by gating on CD4^+^ cells by intracellular staining using flow cytometer. It was observed that there is increase in IFN-γ as the parasitic burden increases till 10th day post infection, which is significantly high as compared to 3rd day post infection in *P. berghei* and *P. yoelii* 17XL. A similar trend was found during the course of infection of *P. chabaudi* and *P. yoelii* 17XNL (Fig. 3A, B).

**Fig. 4:**
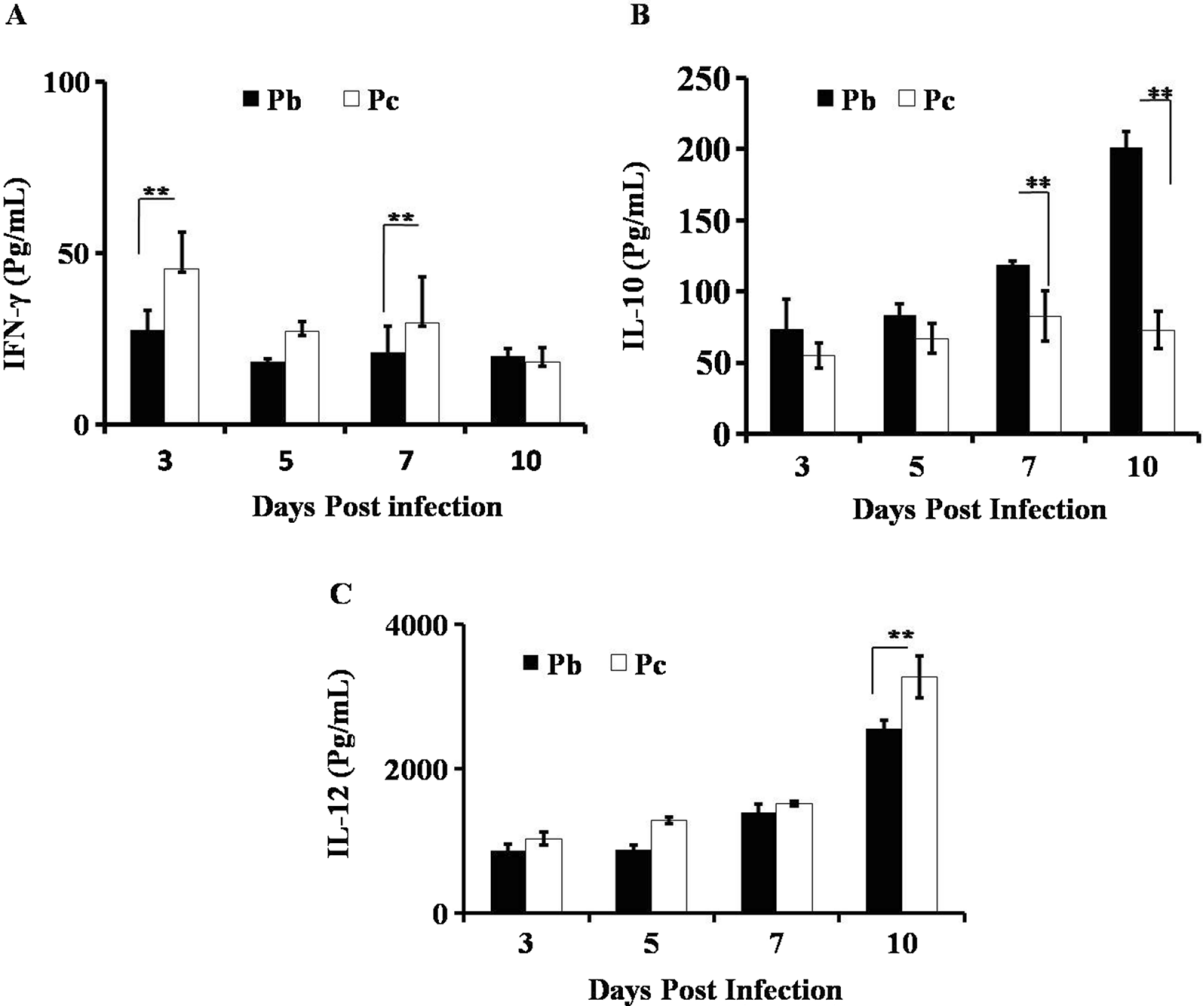
Profile of pro inflammatory and anti-inflammatory cytokine in serum: IFN-γ, IL-10 and IL-12 cytokines secreted in serum by various immune cells during infection with *Plasmodium* (lethal or non-lethal) at 3, 5, 7 and 10 days post infection by using Luminex micro‐bead array system. Data is shown from one of the three independent experiments and statistical significance was determined by Student’s *t *test. **p  < 0.001

Interestingly, when serum concentration of IFN-γ cytokine was determined in lethal and non-lethal strains of parasite by Luminex, a similar pattern was observed as obtained by flow-cytometry. In *P. chabaudi* infected host, initially it was observed that there is higher level of IFN-γ which decreases further with the rising parasitaemia and similar pattern was observed in *P. berghei* infection (Fig. 4A). Next, interleukin-10 (IL-10) production was observed by Luminex at 3rd and 10th day post infection to gain insight into the immune dynamics. A drastic increase in IL-10 producing CD4^+^ T cells was observed at the early stage as well as at later stage of infection with lethal malaria parasites, while with the non-lethal malaria parasites the frequency of IL-10 producing CD4^+^T cells was significantly less **(**Fig. 3C, D). Further, it was also observed that production of IL-10 during entire time of infection remains high in mice infected with lethal (*P*. *berghei* and *P*. *yoelii* 17XL) that might correlate with increased parasitaemia and haemozoin accumulation (Fig. 1E). On the other hand, the level of IL-10 production was low throughout the infection with *P. chabaudi* as in accordance with low parasitaemia **(**Fig. 4B).

The production of IL-10 can be counterbalance by the production of IL-12 during malaria infection. In contrast to above observation, it was noticed that the pro-inflammatory cytokine, IL-12, increases more rapidly in non-lethal parasitic infection as compared to the lethal strain and remained significantly higher during non-lethal infection in comparison to the lethal infection (Fig. 4C).

## Discussion

The outcome of any disease is believed to be dependent on innate and adaptive immune response of host. During malaria infection, the parasite manipulates both wings of immune system to determine their resistance/susceptibility. To have effective anti-malarial response, T-cell activation is important. Expression of inducible co-stimulatory molecules (ICOS) on the surface of T-cells represents a significant component of the T-cell activation as reported in several studies [10, 12, 34]. Recently it has been reported that ICOS is also involved to maintain immune regulation by effective induction of IL-10 [35–38].

Here, the immune response elicited during infection by lethal (*P. berghei* and *P. yoelii* 17XL*)* and non-lethal (*P. chabaudi* and *P. yoelii* 17XNL) rodent malaria parasite was compared. It was revealed that ICOS expression was induced during early time point on 3rd day post infection in the non-lethal as compared to the lethal, while ICOS expression was exacerbated (~ 5–15-fold) mainly during the later stages of disease progression in lethal malaria parasite. Regulatory T cells are known to inhibit inflammatory immune responses and moderate immune pathology [39, 40]. Role of regulatory T cells during malaria infection can be either favourable or harmful for the successful development of diseases. Several groups have reported that depletion of regulatory T cells protect the animals from disease pathogenesis of *P. yoelii* and protects from cerebral malaria in case of *Plasmodium falciparum* [41–44]. Furthermore, other groups have shown that the depletion of regulatory T cells was not able to change parasitic load resulting in mortality [45–47]. These contrasting observations are likely due to the fact that role of regulatory T cell depends upon the host-parasite interaction. Experiments with lethal/non-lethal parasite revealed a new subset of regulatory T-cells (CD4^+^ICOS^+^Foxp3^+^) having role in resistance/susceptibility. A remarkable increase in the expression of CD4^+^ICOS^+^Foxp3^+^ regulatory T cells on 10th day post infection was observed during lethal (*P. berghei* and *P. yoelii* 17XL*)* infection. On the contrary, infection with non-lethal (*P. chabaudi* and *P. yoelii* 17XNL*)* fails to induce significant regulatory T cells. Thus, these data suggest that regulatory T cells induced during infection might help to suppress host immune response leads in unhidden growth of malaria parasite.

The outcome of *Plasmodium* infection is also determined by the fine balance between pro- and anti-inflammatory immune response. Several studies using different mouse models of malaria indicate that IL-10 is accountable for the dynamic growth of parasites and also associated with disease pathogenesis [48–50]. Studies on the role of IL-12 on disease pathogenesis/protection have reported mixed results. Study on non-lethal parasitic infection has demonstrated the protective role of IL-12 against blood stage [51, 52]. Interestingly, another study also supports the fact that the production IL-12 is associated with the pathogenesis of lethal malaria parasite [30, 53].

The present investigation shows a rapid increase of IL-10 by lethal malaria parasite was observed and that may be indicative of immune suppression leading to increased parasitaemia and host mortality. However, in case of infection by non-lethal (*P. chabaudi *and* P. yoelii* 17XNL), the expression of IL-10 is very low during early stage of infection giving ample opportunity for effective immune activation and leading to parasite clearance. Later increase in IL-10 in this case may act as feed-back mechanism to down-regulate the excessive immune activation and maintain homeostasis.

The pathogenic or protective role of IL-12 depends on the immune response elicited by the host-parasite interaction that ultimately determines the outcome of the diseases. For instance, a gradual increase in IL-12 production during lethal infection helps in malaria pathogenesis rather than protection. While in non-lethal parasitic infection, significant change in production of IL-12 production was observed only on day 10th post infection that might be helping in continuous gradual decrease of parasitic load. The contrast in immune response induced depends on several factors affecting the microenvironment including the site of IL-12 production, level of cytokine produced and time of production.

This study demonstrates that the blood stage malaria infection by *P. berghei* modulates expression of various molecules that are associated with activation of immune cells of adaptive immunity. Here, current study perceives that CD4^+^ICOS^+^Foxp3^+^ cells, novel subsets of Treg cells are induced to counterbalance the immune response which are responsible for the production of copius amount of IL-10. Most likely, this may result in immune suppression in lethal *P. berghei* and *P. yoelii* 17XL infection. On the other hand, a smaller number of CD4^+^ICOS^+^Foxp3^+^ cells and reduced amount of IL-10 were observed during the infection with *P. chabaudi* and *P. yoelii* 17XNL.

## Conclusion

In conclusion, present study reveals that high levels of CD4^+^ICOS^+^Foxp3^+^ cells and IL-10 during the course of infection with *P. berghei* and *P. yoelii* 17XL suppresses immune system of host that might help parasite to grow faster, however there is no significant expression of these highly immunosuppressive cells in *P. chabaudi* and *P. yoelii* 17XNL infection. Overall, it is demonstrated that CD4^+^ICOS^+^Foxp3^+^ cells play an important role in regulating immune response of host that ultimately determines the outcome of the disease. Further investigations and innovative strategies are required to elucidate the molecular mechanism responsible for this distinct role of lethal and non-lethal parasite in immuno-modulation.

## Supplementary Information

**Additional file 1: Figures S1, S2.** Expression of Inducible co-stimulatory marker in malaria infection with lethal and non-lethal stain of Plasmodium: mice were infected with 5 × 10^5^ *P. berghei*, *P. chabaudi*, *P. yoelii* 17XL and *P. yoelii* 17XNL parasitized erythrocytes via intra-peritoneal injection. **S1** Splenocytes from infected and control mice were harvested and were stained with antibodies against CD4, and ICOS on 3rd, 5th,7th and 10th-day post infection of *P. berghei* and *P. chabaudi*. **S2** Splenocytes from infected and control mice were harvested and were stained with antibodies against CD4, and ICOS on 3rd, 5th, 7th and 10th-day post infection of *P. yoelii* 17XL and *P. yoelii* 17XNL. Data is shown from one of the three independent experiments consisting of three mice in each group.

## Data Availability

Not applicable.

## Abbreviations

Treg: Regulatory T cell
IL-10: Interleukin-10
ICOS: Inducible co-stimulator
FoxP3: Forkhead box P3
