# Supplementary figures and images for: CD4+ICOS+Foxp3+: a sub-population of regulatory T cells contribute to malaria pathogenesis
Source: Malar J. 2022 Feb 2;21:32. doi: 10.1186/s12936-022-04055-3 (PMC8812217; doi:10.1186/s12936-022-04055-3)

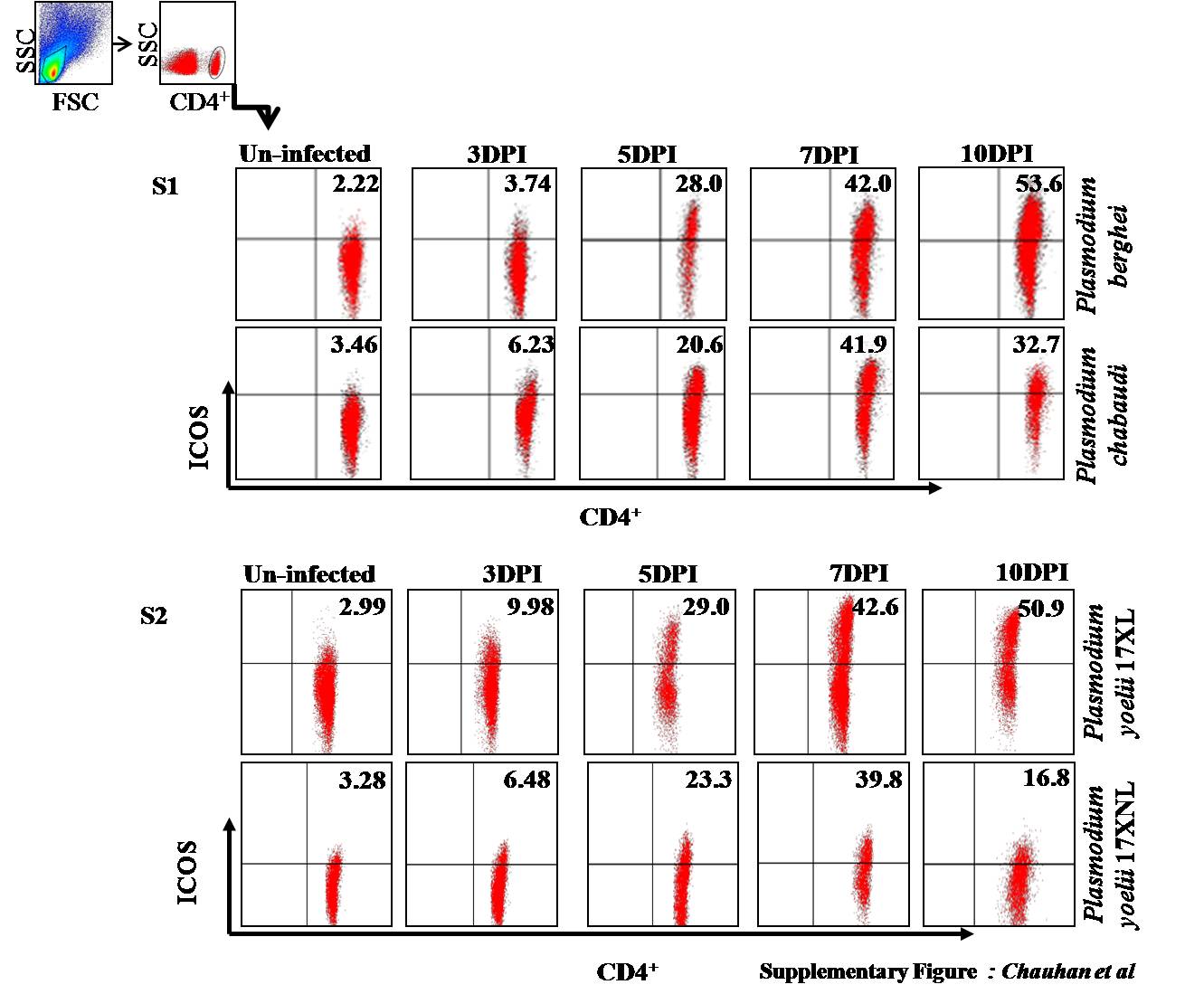

Supplement: Supplementary file 1 — Additional file 1: Figures S1, S2. Expression of Inducible co-stimulatory marker in malaria infection with lethal and non-lethal stain of Plasmodium: mice were infected with 5 × 10^5^ P. berghei, P. chabaudi, P. yoelii 17XL and P. yoelii 17XNL parasitized erythrocytes via intra-peritoneal injection. S1 Splenocytes from infected and control mice were harvested and were stained with antibodies against CD4, and ICOS on 3rd, 5th,7th and 10th-day post infection of P. berghei and P. chabaudi. S2 Splenocytes from infected and control mice were harvested and were stained with antibodies against CD4, and ICOS on 3rd, 5th, 7th and 10th-day post infection of P. yoelii 17XL and P. yoelii 17XNL. Data is shown from one of the three independent experiments consisting of three mice in each group. [file 12936_2022_4055_MOESM1_ESM.jpg]
